# Clinical Comparison of CAD/CAM vs. KLS L1^®^ Mandible ReconGuide in Fibula Free Flap Mandible Reconstruction: A Retrospective Clinical Study

**DOI:** 10.3390/jcm14030736

**Published:** 2025-01-23

**Authors:** Lena Denk, Anna-Maria Sigwart, Andreas Kolk, Benjamin Walch

**Affiliations:** 1 Department of Oral and Maxillofacial Surgery, Medical University of Innsbruck, A-6020 Innsbruck, Austria; 2 Department of Oral and Maxillofacial Surgery, Medical University Salzburg, A-5020 Salzburg, Austria

**Keywords:** reconstructive surgical procedures, mandibular reconstruction, microsurgery, free fibula flap, surgical equipment, cutting guide, computer-aided design, computer-aided manufacturing, postoperative complications, oropharyngeal dysphagia, oral cancer

## Abstract

**Introduction**: The fibula free flap (FFF) is regarded as the workhorse for lower jaw reconstruction in maxillofacial surgery. Imitating the preexisting shape of the mandible by an FFF while meeting various clinical and geometric aspects can be challenging, even for an experienced surgeon. To enhance the quality and reproducibility of the reconstruction process, several tools are available, mainly based on CAD/CAM techniques and the KLS L1 Mandible ReconGuide. The objective of this study was to examine the clinical use of the KLS L1^®^ Mandible ReconGuide compared to CAD/CAM templates. **Material and Methods**: In this study, we compared 25 patients who underwent mandibular reconstruction by a FFF with either the KLS L1^®^ Mandible ReconGuide (G1, *n* = 17) or personalized CAD/CAM-based cutting guides (G2, *n* = 8). We performed a pre- and postoperative 3D image reconstruction using standard triangle language (STL) to quantify the anatomical results in terms of volume deviations, intercondylar distance, and gonial angle, as well as clinical criteria such as surgery time, function, and postoperative complications. **Results**: The analysis of pre- and postoperative clinical outcomes in 25 patients revealed no statistically significant differences between the groups. However, it was observed that longer surgery time was group-independent, associated with a 5.63% increase in the length of hospital stays (*p* = 0.0002). In terms of geometric criteria, the only significant difference referred to the postoperative length of the symphysis, which measured 34.32 mm in G2 versus 34.78 mm in G1 (*p* = 0.046). **Conclusions**: Both the KLS L1^®^ Mandible ReconGuide and CAD/CAM templates seem equivalent, effective devices for standardized mandibular reconstruction, with their suitability depending on the specific indications and the segments involved.

## 1. Introduction

Since its first description by Hidalgo in 1989 [1], the fibula free flap (FFF) has become the workhorse for mandibular reconstruction. It is currently the most widely used flap for this application and is considered the gold standard worldwide. This FFF offers numerous advantages, including long donor vessels, good bone quality, morphology closely resembling the mandible, good perfusion of the skin paddle, the double-barrel technique to gain additional height for dental implant placement, and the capability to reconstruct the entire mandible if necessary [2,3].

Raising an FFF in the correct dimensions and cutting it at the appropriate angles to ensure a proper fit in the mouth while accurately recreating the original shape can be quite challenging, especially when multiple segments are involved. However, there are various options and tools available that can simplify this process, leading to a more precise outcome compared to freehand methods [4,5,6,7].

Nowadays, CAD/CAM technologies are often regarded as the gold standard for mandibular reconstruction [6,7]. The CAD or Virtual surgical planning phase allows for the precise recreation of the damaged mandible’s form and facilitates the identification of the optimal fibula harvest site. Additionally, the shaping of individual fibular segments to accurately replicate the outer contour of the mandible can be performed virtually, streamlining steps that would otherwise need to be executed manually by the surgeon. One option is to utilize digital planning to outline the surgical cuts and reconstruction in advance, followed by printing a template for cutting both the mandible and accordingly the fibula. The planning and printing processes can be conducted in-house or in collaboration with specialized external companies. Also possible, the surgeon may handle the planning in-house while the production of the templates is outsourced to an external company [6,7,8].

Since the late 1980s, virtual surgical planning, CAD/CAM-guided surgery, and patient-specific implants have been extensively utilized, greatly advancing maxillofacial surgery. CAD/CAM-guided mandible reconstruction results in improved mouth opening, reduced postoperative pain, enhanced bony consolidation, esthetic and less midline deviation, and shorter hospitalization duration and surgery time [6,8,9].

As an alternative to the CAD/CAM custom templates the KLS L1^®^ Mandible ReconGuide (KLS Martin SE & Co KG, Tuttlingen, Germany) is offered as a versatile tool designed for the reconstruction of up to three-segment of the horizontal part of the FFF. This tool is non-individualized, can be sterilized, and is reusable, similar to other surgical instruments. The system consists of two cutting guides: one for the fibula and one for the mandible. The mandible guide is color-coded for the left and right sides of the lower jaw, with corresponding colors on the fibula template. Each cutting guide is made up of three components, allowing for the reconstruction of up to a three-segment FFF. The segments are numbered to ensure proper alignment during reconstruction. 

The lateral segments, which correspond to the corpus in cases of a three-segment FFF, can be adjusted in length. However, in contrast to the CAD/CAM procedure, the length of the central part—the symphysis—as well as the angle of the latter to the lateral segments are fixed in the KLS L1^®^ Mandible ReconGuide. These standardized measurements are designed to approximate the average values for the population [10].

The aim of these tools is twofold: to streamline the surgical process and reduce surgical time, while also ensuring more precise results with minimized intersegmental distance, leading to improved healing outcomes. Additionally, they strive to achieve postoperative results that closely resemble the original mandible, thereby enhancing functional rehabilitation, alignment of the soft tissue, and, ultimately, aesthetic outcomes [11,12].

Extended operation times are linked to a higher incidence of flap failure and overall higher complication rates encompassing deep venous thrombosis, pulmonary embolism, ventilation problems, wound healing disturbances, infection, and postoperative delirium [13,14].

While a patient-specific reconstruction using CAD/CAM technology may seem to be inherently superior, there are also drawbacks to CAD/CAM-planned reconstruction. These include the time required for planning if completed in-house, the higher costs associated with custom cutting guides when planning is outsourced, and the reduced flexibility in terms of the requirement of additional resections in cases of histologically proven closed margins or other complications during surgery that necessitate different resection than initially planned [15,16].

The aim of this study was to investigate, whether there were differences in intraoperative handling, patients‘ complication rates, and the postoperative outcome depending on the method used for reconstruction: personalized CAD/CAM templates versus the KLS L1^®^ Mandible ReconGuide.

## 2. Material and Methods

For this retrospective cohort study, approval was obtained on 5 April 2024 from the ethics committee of the Medical University of Innsbruck. (Approval number 1063/2024) The research was conducted in accordance with the Declaration of Helsinki.

The study was conducted at the Department of Maxillofacial Surgery at the University Clinic in Innsbruck and comprised 25 patients who received an FFF for lower jaw reconstruction between 2019 and 2024. Written informed consent was obtained from all subjects involved in the study.

The inclusion criteria were as follows: Patients at the Department of Maxillofacial Surgery at the University Clinic in Innsbruck who underwent reconstruction of the lower jaw using an FFF between 2019 and 2024. Participants had to be at least 18 years old and must have had imaging with tomographies conducted both before and after surgery, along with documentation regarding the reconstruction tool used.

The exclusion criteria included the reconstruction of the joint itself, as intraarticular surgery significantly affects the function and movement of the jaw compared to reconstructions exclusively limited to the horizontal parts of the mandible.

The patients were divided into two groups, based on whether CAD/CAM-planned personalized cutting guides (G2) or the KLS L1^®^ Martin ReconGuide (G1) ultimately were used for the reconstruction. Additionally, we categorized the patients due to the indication for FFF reconstruction, which included cancer, medication-related osteonecrosis of the jaw, or osteoradionecrosis, in order to assess whether there were significant differences in postoperative outcomes and complications.

The division of mandibular segments has been categorized according to Urken’s Classification, which divides the mandible based on functional parameters. Urken classifies the mandible into four segments: the condyle (C), the ramus (R), the body (B), and the symphysis (S). While involvement of the condyle affects postoperative masticatory function, resections closed to the ramus disrupt the masticatory sling. Reconstruction of the symphysis, on the other hand, typically requires detaching a significant portion of the suprahyoid muscles [17].

### 2.1. Preoperative Planning

All patients undergoing an FFF had a planning computer tomography (CT) scan of the head and both legs to confirm a three-vessel situation in the lower leg, ensuring adequate nourishment for the donor site after removing the peroneal vessels while harvesting the FFF.

For cases utilizing CAD/CAM technology, this imaging was employed for pre-surgical planning and uploaded to the KLS Martin planning platform. During a collaborative meeting, we discussed the cutting regions, potential vessel connections, and dimensions of the flap. Subsequently, the template planning and construction were carried out off-site by KLS Martin, and we received the sterilized templates in the week of the surgery.

With the use of KLS L1^®^ Mandible ReconGuide, no preoperative planning sessions were necessary, and the KLS L1^®^ Mandible ReconGuide, along with other instruments and plates, was readily available on the day of the operation.

### 2.2. Operative Procedure KLS L1 Mandible ReconGuide

**Figure 1:**
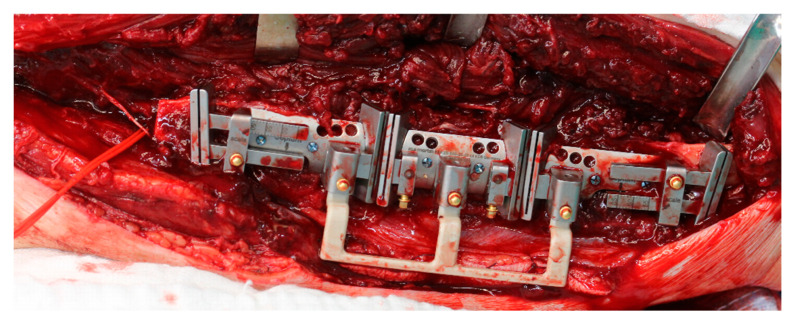
KLS L1^®^ Mandible ReconGuide placed on the fibula of study patient.

During the operation, the KLS L1 Mandible ReconGuide cutting guides were securely attached to the fibula after identifying the length of the required segments at the mandible. Contrary to individualized templates, which provided a precise fit, the ReconGuide allowed more flexibility in placement on the fibula. Once fixed with screws, cutting was primarily performed using a surgical saw (Figure 1).

**Figure 2:**
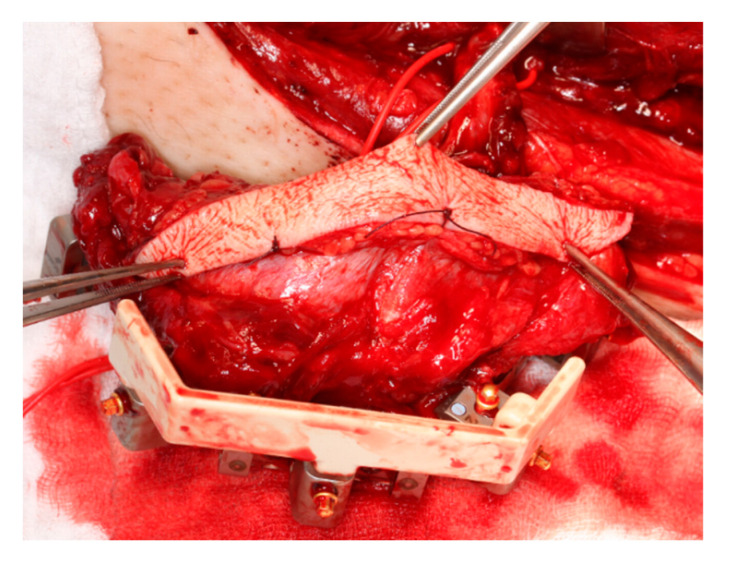
Same case as Figure 1 after fixation bracket brought onto the KLS L1^®^ Mandible ReconGuide.

**Figure 3:**
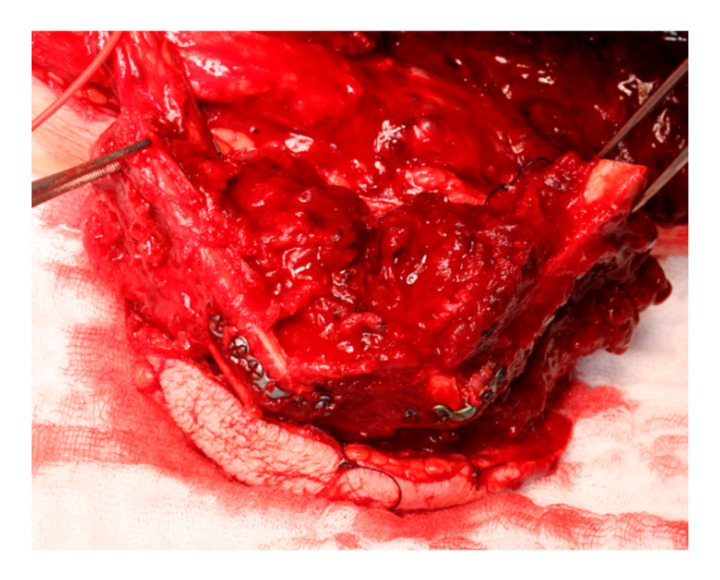
Same case as Figure 4 after fixation with pre-bended miniplates in the same case as Figure 1.

To facilitate a jaw-like reconstruction from the straight fibula, KLS Martin offers a fixation bracket that integrates with the KLS L1 Mandible ReconGuide cutting guide, along with plates set at the appropriate angles (Figure 2 and Figure 3).

The KLS L1^®^ Mandible ReconGuide system also includes cutting guides for the mandibular resection that are compatible with the templates for the FFF, ensuring a precise fit for the transplant. After the FFF was transformed into the new mandible and secured at the resection site, microsurgical anastomosis was performed, followed by the reconstruction of soft tissues with adaption of muscles.

### 2.3. Operative Procedure CAD/CAM

All our CAD/CAM cases were outsourced for planning to specialized external companies. The templates were tailored to the corresponding bone by contouring them to the shape of the fibula and identifying anatomical like cortical features, teeth, the symphysis, or angle, which were determined in the planning session. Once planning was complete, the templates for mandibular resection, fibula free flap (FFF), and patient-specific osteosynthesis plates were shipped to our department.

**Figure 4:**
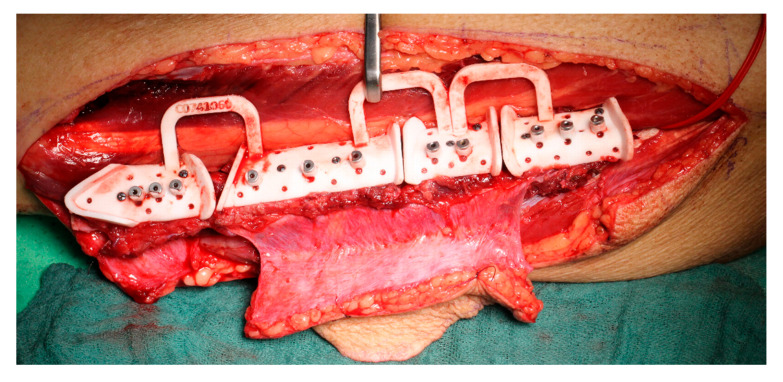
CAD/CAM planned cutting guide placed on the fibula of study patient.

Upon arrival, the materials were sterilized. Intraoperatively, the anatomical landmarks and features were identified aided by supplementary printed 3D models. After verifying proper alignment with the bone, the templates were secured using screws (Figure 4). Resection was then performed using guide slots and edges, following a process similar to the KLS L1^®^ Mandible ReconGuide cases.

**Figure 5:**
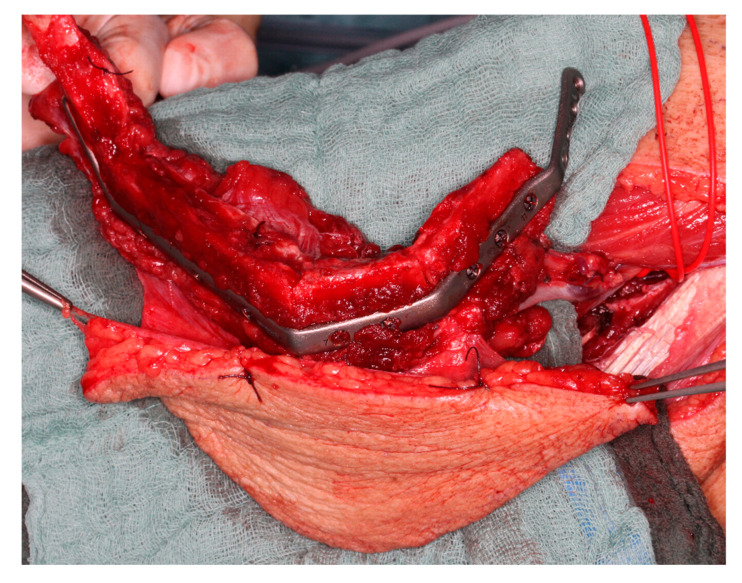
Same case as Figure 4 after fixation with preoperative planned plate.

When utilizing CAD/CAM technology, fixation plates were either manually bent, or individualized plates were pre-ordered. Reconstruction templates or the patient-specific load-bearing plates themselves were used to shape the fibula segments into the new mandible (Figure 5).

### 2.4. Data Collection and Processing

To compile all cases of mandible reconstruction using a microvascular fibula graft, we selected all operations conducted between 2019 and 2024 based on the ICD-10 Codes K10.2 and C97, and MEL Codes HA020, QZ080, HA050, and QA060, utilizing Torin (Getinge IT Solutions GmbH, Version 24.2.0.3., Getinge, Sweden), our operation management tool.

For clinical data, including age, sex, indications, and intra- and postoperative complications such as thrombosis, reintervention, infection, flap failure, and plate exposure, we used the KIS PowerChart System (Cerner Corporation, Version 2018.01, Kansas City, MO, USA). The collected data were then transferred to Microsoft Excel (Microsoft, Version 2409, Redmond, Washington, USA).

Pre- and postoperative imaging was compared for each patient using 3D models. To do this, we exported digital imaging and communications in medicine (DICOM) data from computed tomography and digital volume tomography before and after reconstruction using Syngo.Share View (Siemens Healthineers, Release VA32C, Munich, Germany), the radiology program employed at the Medical University of Innsbruck.

Next, data were imported into Mimics (Materialise, Version 24.0, Leuven, Belgien), which has been used to create standard triangle language (STL) 3D models of both the lower jaw and the entire face for geometric measurement.

**Figure 6:**
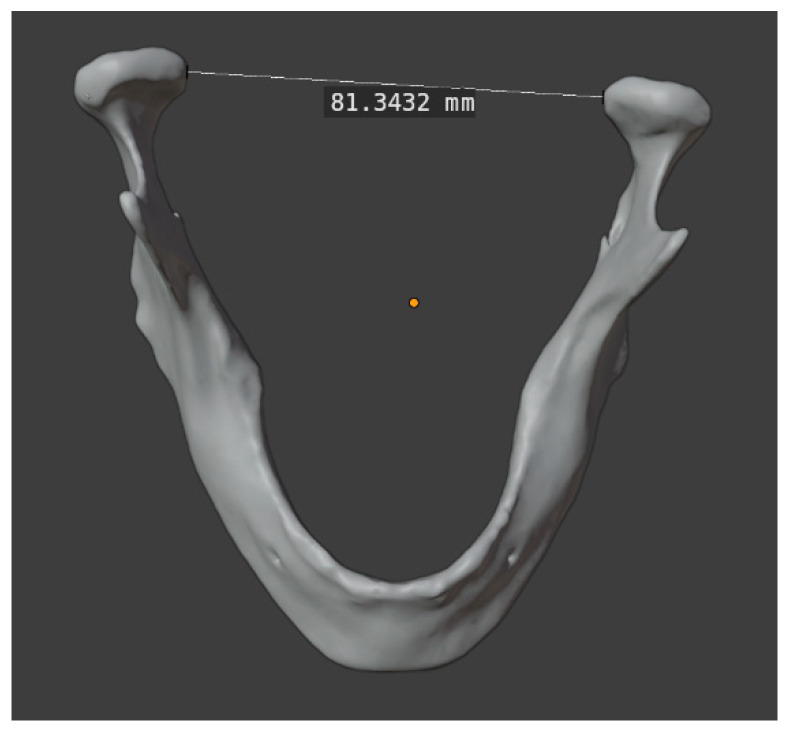
Measuring the intercondylar distance using Blender in a preoperative 3D model.

**Figure 7:**
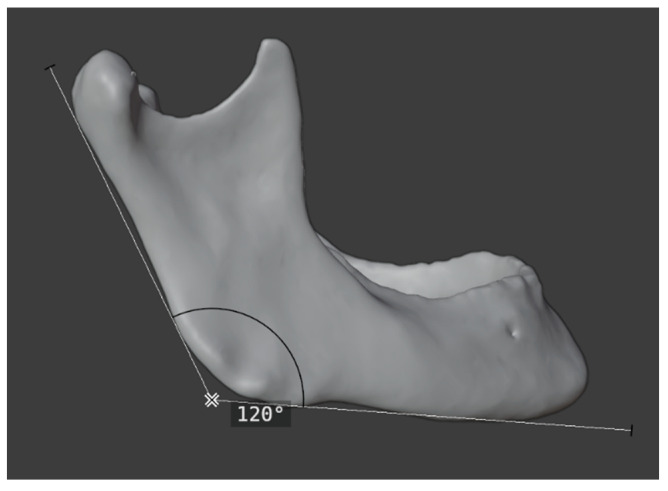
Measuring the gonial angle using Blender in a preoperative 3D model.

For the latter, Blender (Blender, Version 4.2, Amsterdam, Netherlands) was applied. The parameters assessed included intercondylar distance, gonial angle, intersegmental distance, and length of symphysis. (Figure 6 and Figure 7).

The intercondylar distance was defined as the linear distance between the two most medial points on the mandibular condyle. To measure the gonial angle, two tangents were constructed, one each touching the ramus and the body of the original or reconstructed mandible at the outermost posterior and the other at the most caudal points. The angle formed by the intersection of these tangents was then measured.

The symphyseal length was measured by defining the symphysis segment according to Urken’s classification [17]. This segment is located between the two canines, which were identified clinically. In edentulous cases, the location was approximated by measuring one premolar width mesially from the mental foramen.

Postoperatively, the reconstructed symphyseal length of the fibula free flap (FFF) was defined as the longest distance along this segment. Measurements were taken on the buccal side, as this segment is inherently longer there.

In addition, gaps between the reconstructed segments were assessed. The intersegmental distance was defined as the widest linear gap between adjacent segments following reconstruction.

### 2.5. Statistical Analysis

Patient demographics and clinical characteristics were summarized using descriptive statistics. Continuous variables are reported as mean and standard deviation (SD) or median with interquartile range (IQR) as appropriate. Categorical variables are presented as counts and percentages. Group comparisons were conducted using Mann–Whitney U test for continuous variables and Fisher’s exact test for categorical data. Log-binomial regression and Poisson regression were used to examine the association of duration of surgery with delirium and length of stay, respectively. Data were analyzed using R (R Foundation for Statistical Computing, Version 4.4.2, Vienna, Austria).

## 3. Results

### 3.1. Clinical Criteria

In total, we identified 25 patients who met the complete inclusion criteria. Among these, sixteen were male and nine were female. Eighteen patients (eleven males and seven females) underwent reconstruction using the KLS L1^®^ Mandible ReconGuide (Group 1—G1), while seven patients (five males and two females) received digitally planned CAD/CAM templates (Group 2—G2).

In terms of clinical criteria, no statistically significant differences were obtained between the groups (G1 vs. G2) (Table 1).

#### 3.1.1. Operative Data

The median operation time in G1 was 12.28 [IQR 10.50, 13.49], while in G2, it was 11.87 [IQR 10.70, 16.10] minutes, indicating no significant difference (*p* = 0.607). The duration of hospitalization ranged from 13 to 47 days (IQR 23.50 [19.25, 32.00]) in G1 and from 14 to 60 days (39.00 [IQR 24.00, 42.50]) in G2 (*p* = 0.226).

#### 3.1.2. Postoperative Complications

Postoperative delirium developed in six patients, with four in the KLS L1^®^ Mandible ReconGuide group and two in the CAD/CAM group. For each additional hour of surgery, the risk of delirium increases by 7.78%, though this association was not statistically significant (*p* = 0.694). In contrast, for every 1 h increase in surgical duration, the length of stay increases by 5.63%, a finding that is statistically significant (*p* = 0.00024)

There were no instances of flap failure in either group.

However, reintervention was necessary in ten cases, with eight of these patients having undergone reconstruction with the KLS L1^®^ Mandible ReconGuide. Most procedures were performed to correct minor necrotic lesions or for cosmetic purposes. Reintervention to address dehiscences was necessary only twice—once for a patient in Group G1 and once for a patient in Group G2.

Wound dehiscence occurred in eleven patients, six of whom exhibited plate exposure during postoperative evaluations—four in the KLS L1^®^ Mandible ReconGuide group and two in the CAD/CAM group.

Notably, none of the patients experienced postoperative thrombotic incidents.

Infections were reported in nine patients, evenly distributed between the two groups. Four infections were putrid bacterial infections localized at the reconstructed mandible, one at the donor site of the fibula, and four patients experienced systemic respiratory infections, primarily pneumonia.

#### 3.1.3. Functional Rehabilitation

**Figure 8:**
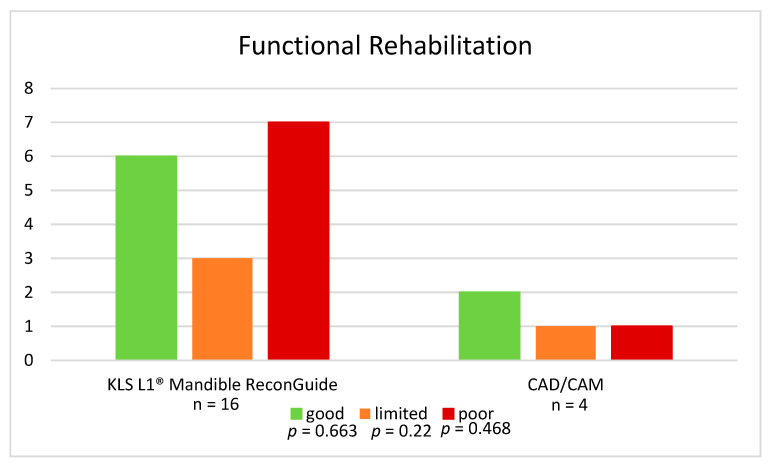
Functional rehabilitation KLS L1^®^ Mandible ReconGuide vs. CAD/CAM.

Regarding chewing function post-surgery, we assessed functional rehabilitation in both groups. Orofunctional rehabilitation levels of 20 patients could be evaluated: 16 in the KLS L1^®^ Mandible ReconGuide group and 4 in the CAD/CAM group. Overall, eight patients demonstrated good rehabilitation (six in the KLS L1^®^ Mandible ReconGuide group and two in the CAD/CAM group), while an equal number (seven in the KLS L1^®^ Mandible ReconGuide group and one in the CAD/CAM group) exhibited poor dental function or no rehabilitation remaining dependent on a PEG tube for nutrition. Additionally, four patients (three from the KLS L1^®^ Mandible ReconGuide group and one from the CAD/CAM group) showed limited functional rehabilitation, struggling with chewing and swallowing foods of higher IDDSI scale consistencies. All of these show no statistically significant differences (Figure 8).

### 3.2. Geometric Criteria

For evaluation of anatomical restoration of the resected part of the mandible, geometric criteria were quantified, in particular intercondylar distance, gonial angle, intersegmental distances, and the lingual length of the symphysis (Table 2).

#### 3.2.1. Intercondylar Distance

Regarding geometric criteria, our study found no statistically significant differences in intercondylar distance between the reconstruction methods.

Both groups exhibited an intercondylar distance of 84 mm prior to resection and 85.03 [IQR 79.43, 93.91] mm in G2 and 85.50 [IQR 81.57, 88.04] mm in G1 after replacement by the FFF, indicating a non-significant transversal intercondylar expansion in both groups (*p* = 1.000).

The only statistically significant difference observed was related to the involvement of the Urken segments. If the symphysis (S) segments are affected, the resulting intercondylar distance becomes significantly smaller compared to the situation, if only the ramus (R) or body (B) are involved. Notably, this difference is a general association and is not specific to any particular template type. Since the ReconGuide is not suitable for reconstructing the ascending ramus or the condylar head, patients with condyle involvement had exclusively been treated with CAD/CAM planned templates and had been excluded from these series.

#### 3.2.2. Gonial Angle

The gonial angles, defined as the angle between the base of the mandibular body and the tangent at the ascending ramus, showed minimal differences between the postoperative outcome (mean 127.00° G1, 123.00° G2) and the initial measurements (mean 121.50° G1, 125.00° G2), with no significant difference between the two groups (*p* = 0.832). On average, the gonial angle decreased by −3.00° [IQR −6.50°, 4.00°] for G2 and −2.00° [IQR −10.25°, 2.50°] for G1, following surgery. Although some individual cases demonstrated a difference of up to 29° between pre- and postoperative data, this variation was not statistically significant (*p* = 0.879).

#### 3.2.3. Intersegmental Distances

Patients in neither group showed any relevant gaps between the reconstructed segments as a potential base of bone healing disturbances, therefore the intersegmental distances for all patients were 0 mm.

#### 3.2.4. Length of Symphysis

The postoperative length of the symphysis showed a statistically significant difference between G1 and G2. 

The symphysis length in the CAD/CAM group was 34.78 mm, while in the ReconGuide group, it measured 30.41 mm (*p* = 0.046). However, there was no significant difference in the pre- and postoperative changes in symphysis length between the two groups.

## 4. Discussion

Currently, there is a limited amount of data about this topic. Weitz et al. reported similar outcomes with only minor differences between the two FFF segmentation methods in their study, comprising 40 patients [18].

We observed low complication rates irrespective of the applicated method, suggesting that the KLS L1 ReconGuide and CAD/CAM planned templates are effective tools for the reconstruction of the horizontal tooth-bearing area of the mandible, contributing to quality surgical care.

It is important to carefully consider the indications for each cutting guide method. The ReconGuide offers the advantage of faster utilization, as it requires no preoperative planning, and is effective for reconstructions involving up to three FFF segments. However, it is limited to the use in segments S, B, and parts of R, and cannot be applied for capitulum or ascending ramus reconstruction [19]. In contrast, CAD/CAM requires more upfront planning but can be applied to any mandibular defect and location, including the condyle. Additionally, CAD/CAM is versatile, enabling its use for other microvascular bone transplants, such as scapula or iliac crest grafts. On the other hand, CAD/CAM templates have fixed anatomical positions and predefined cutting areas, making it nearly impossible to adjust cutting positions due to preoperative unexpected more extended resection requirements [16]. As a result, the ReconGuide was used in most patients in this study who required immediate reconstruction due to a malignant tumor.

As highlighted earlier, CAD/CAM templates rely obligatory on CT imaging, which introduces several challenges. One significant issue is the difficulty in ad hoc adjusting the extent of resection intraoperatively. Additionally, the planning process is highly sensitive to artifacts caused by metallic foreign objects such as plates from previous surgeries, dental restorations, or implants. These artifacts can compromise image quality, making it challenging to precisely design the templates and ultimately affecting their fit during surgery.

Furthermore, while a CT with 3 mm slices is sufficient for evaluating vessel anatomy at the neck and tumor extent, CAD/CAM template planning requires fine-slice CT imaging, preferably with 0.8–1 mm slices. This higher resolution increases the radiation dose, ref. [20] a particularly important consideration, especially for younger patients undergoing reconstruction due to cysts, malformations, or syndromes.

Another important factor is cost. CAD/CAM templates are custom-made for each patient and cannot be reused, increasing overall expenses. Ref. [21] In contrast, the KLS L1^®^ Mandible ReconGuide cutting guide offers a reusable alternative; it can be sterilized and utilized in future reconstructions, thereby reducing costs and enhancing efficiency.

The use of CAD/CAM templates also demands extensive preoperative planning time [22] and also requires effort from surgeons. They must meticulously design forecasted resection lines in advance, taking into account the limitations of modifying the resection area intraoperatively. This planning process often involves several hours of collaboration between the surgical team and external providers.

Nevertheless, while the ReconGuide is often considered more time-efficient than CAD/CAM templates, this advantage holds primarily in terms of preoperative planning [10]. However, based on our findings, neither option can be definitively deemed more time-saving during the actual procedure. Thus, as the surgery duration is comparable for both proceedings, neither method can be preferred solely based on anesthesia duration, which helps minimize the complication rate and, thus, the length of intensive care unit stays as well as the time of total hospitalization [23].

Time constraints in preoperative planning can significantly affect oncologic patients. Beyond the planning itself, additional time is required for the manufacturing, delivery, and sterilization of custom templates. This creates a prolonged interval between initial diagnosis and surgery, which may have critical implications for patient outcomes in case of primary tumor resections [21]. Prolonged planning may allow tumor progression, making the surgery more complex, reducing the chances of successful outcomes, and ultimately worsening patient survival rates [24].

Oral rehabilitation was evaluated through eating capabilities, as restoring oral function is one of the primary goals of this surgery [25]. We did not examine the type of dental rehabilitation in this study. However, existing studies suggest that factors such as prosthetic rehabilitation, as well as the location and number of dental implants, play a critical role and may influence long-term outcomes [26,27,28]. Studies have demonstrated that CAD/CAM reconstruction achieves favorable functional outcomes [29]. We observed comparable results for both CAD/CAM and KLS L1^®^ Mandible ReconGuide, though our findings are based on a limited number of patients.

The average value for the gonial angle in other studies is reported to be between 115 and 128°, depending on the patient’s age [30,31,32,33]. In our study, the mean postoperative gonial angle was consistent with this benchmark, leaning towards the higher end of the range. This finding suggests that both reconstruction options are suitable for mandible reconstruction.

The intercondylar distance was reported as between 98 [34] and 126 mm, each measured from the centers of the condyles [35]. In our study, we measured the distance between the medial poles of the condyles like Hackney et al. did, measuring 88 mm [36]. We found a mean intercondylar distance of about 85–86 mm for both reconstruction methods.

The KLS L1^®^ Mandible ReconGuide is a standardized guide for reconstruction, whereas CAD/CAM templates are custom-designed for each individual. While the length of the corpus can be individually adjusted, allowing greater flexibility for intraoperative decisions regarding resection, the symphysis remains standardized [10]. As a result, the KLS L1^®^ Mandible ReconGuide is expected to yield more uniform jaw outcomes, with an outer symphysis length of 32 mm [19]. We initially hypothesized that this could lead to a broader symphysis segment, particularly in women, potentially resulting in a more prominent chin that might appear less feminine. However, based on our findings, the outcomes with the KLS L1^®^ Mandible ReconGuide closely align with the preoperative conditions of the symphysis. It is important to note, though, that there were more males than females in both groups, which may influence the interpretation of these results.

The angles at which the lateral segments are fixed to the symphysis are standardized, based on the average measurements from reconstructed patients. This may lead to different values compared to non-reconstructed jaws [19].

Since the physiological jaw has a natural curvature without fixed angles, comparing the postoperative mandibular angles with the preoperative configuration is challenging. To address this, we measured the intercondylar distance, as the angle of the mesial part of the jaw influences the position of the condyles. However, we did not observe significant differences, which could be attributed to compensatory adaptions in these areas between the reconstructed and non-reconstructed parts of the jaw, such as the ascending ramus, which was not reconstructed with the KLS L1^®^ Mandible ReconGuide.

Further studies are needed to determine whether the chin angle in reconstructed mandibles is comparable to that of the physiological mandible.

### Limitations

This study provides valuable insights into the outcomes of mandibular reconstruction using two different methods: ReconGuide and CAD/CAM-guided surgery. However, we acknowledge several limitations inherent to our study design, which should be considered when interpreting the results.

Firstly, as a retrospective cohort study, this research is subject to limitations such as the absence of randomization. The lack of randomization means that patient selection for each reconstruction method may have been influenced by factors not accounted for in the analysis, such as preexisting comorbidities or other confounding variables. These baseline differences between groups could potentially impact the comparability of the outcomes.

Another significant limitation is the unequal sample sizes between the two groups, with 17 patients in the ReconGuide cohort and only 8 patients in the CAD/CAM cohort. This discrepancy in group sizes increases the risk of Type II errors, meaning there is a possibility that true differences between the groups may not have been detected due to insufficient statistical power. Future studies with larger and more balanced sample sizes would be necessary to validate these findings and reduce the risk of such errors.

Additionally, the heterogeneity of the cohort regarding the indications for mandibular reconstruction presents another limitation. Patients in the study were undergoing reconstruction for various reasons, such as mandibular osteoradionecrosis (MORN) and surgical tumor excision. These different indications may influence the outcomes of the procedures, as factors such as tumor size, radiation history, and tissue viability can vary significantly between patients. We have discussed this potential source of variability in our analysis and recognize that it complicates direct comparisons between the two groups.

Despite these limitations, our study contributes valuable data to the field of mandibular reconstruction and provides a foundation for future research aimed at optimizing these techniques.

## 5. Conclusions

Both the KLS L1^®^ Mandible ReconGuide and CAD/CAM planned templates appear to be suitable for mandibular reconstruction using fibula free flaps (FFF). Our data suggests that the clinical and geometric outcomes between the two approaches exhibit only minor differences, which are unlikely to be clinically relevant. Given its cost-effectiveness, reduced reliance on preoperative imaging, and time efficiency in planning and scheduling surgery, the ReconGuide may be a suitable option for mandibular FFF reconstruction, provided that no joint-bearing segments are involved.

However, further studies with larger patient cohorts and prospective, randomized data are necessary to validate these findings and to determine any specific advantages of each approach in terms of clinical outcomes.

## Figures and Tables

**Table 1:** Clinical criteria: share of each group (n + IQR or %).

	KLS L1^®^ Mandible ReconGuide (G1)	CAD/CAM (G2)	*p*-Value
Duration of surgery (min)	12.28 [10.50, 13.49]	11.87 [10.70, 16.10]	0.607
Hospitalization (d)	23.50 [19.25, 32.00]	39.00 [24.00, 42.50]	0.226
Postoperative delirium	4 (22.2%)	2 (28.6%)	1
Thrombosis	0	0	n.a.
Reintervention	8 (44.4%)	2 (28.6%)	0.785
Plate exposition	4 (22.2%)	2 (28.6%)	1
Wound dehiscence	7 (38.9%)	4 (57.1%)	0.706
Infection (local)	3 (16.7%)	2 (28.6%)	0.911
Infection (systemic)	2 (11.1%)	2 (28.6%)	0.644

**Table 2:** Geometric criteria: angles are measured in degrees (°), and distances in millimeters (mm).

	KLS L1^®^ Mandible ReconGuide (G1)	CAD/CAM (G2)	*p*-Value
Intercondylar distance (preop)	83.99 [81.10, 86.93]	84.25 [82.68, 87.36]	0.672
Intercondylar distance (postop)	85.50 [81.57, 88.04]	85.03 [79.43, 93.91]	1.000
Intercondylar distance (difference)	−0.33 [−2.15, 1.57]	−0.78 [−3.54, 2.55]	0.952
Gonial angle (preop)	121.50 [119.25, 129.75]	125.00 [118, 127.50]	0.716
Gonial angle (postop)	127.00 [118.00, 133.00]	123.00 [119, 132]	0.832
Gonial angle (difference)	−2.00 [−10.25, 2.50]	−3.00 [−6.50, 4.00]	0.879
Length of symphysis (preop)	30.93 [28.75, 35.37]	30.86 [28.19, 33.43]	0.641
Length of symphysis (postop)	30.41 [28.95, 32.55]	34.78 [32.75, 35.90]	0.046
Length of symphysis (difference)	−0.40 [−2.25, 2.77]	−1.59 [−7.11, 0.38]	0.205
Intersegmental distance	0	0	n.a.

## Data Availability

The data presented in this study are available on request from the corresponding author due to privacy, legal, and ethical reasons.

## Abbreviations

CAD/CAM	Computer-aided design/computer-aided manufacturing
CT	Computer tomography
FFF	The fibula free flap
IDDSI	International Dysphagia Diet Standardisation Initiative
IQR	Interquartile range
PEG	Percutaneous endoscopic gastrostomy
PSI	Patient-specific implant
SD	Standard deviation
STL	Standard triangle language
